# Non-pharmaceutical prevention of hip fractures – a cost-effectiveness analysis of a community-based elderly safety promotion program in Sweden

**DOI:** 10.1186/1478-7547-6-11

**Published:** 2008-05-30

**Authors:** Pia Johansson, Siv Sadigh, Per Tillgren, Clas Rehnberg

**Affiliations:** 1 Karolinska Institutet, Department of Public Health Sciences, Stockholm, Sweden; 2 Mälardalen University, School of Health, Care and Social Welfare, Västerås, Sweden; 3 Karolinska Institutet, Medical Management Centre, Stockholm, Sweden

## Abstract

**Background:**

Elderly injuries are a recognized public health concern and are due to two factors; osteoporosis and accidental falls. Several osteoporosis pharmaceuticals are considered cost-effective, but intervention programs aiming at preventing falls should also be subjected to economic evaluations. This study presents a cost-effectiveness analysis of a community-based elderly safety promotion program.

**Methods:**

A five-year elderly safety promotion program combining environmental structural changes with individually based measures was implemented in a community in the metropolitan area of Stockholm, Sweden. The community had around 5,500 inhabitants aged 65+ years and a first hip fracture incidence of 10.7 per 1,000 in pre-intervention years 1990–1995. The intervention outcome was measured as avoided hip fractures, obtained from a register-based quasi-experimental longitudinal analysis with several control areas. The long-term consequences in societal costs and health effects due to the avoided hip fractures, conservatively assumed to be avoided for one year, were estimated with a Markov model based on Swedish data. The analysis holds the societal perspective and conforms to recommendations for pharmaceutical cost-effectiveness analyses.

**Results:**

Total societal intervention costs amounted to 6.45 million SEK (in Swedish krona 2004; 1 Euro = 9.13 SEK). The number of avoided hip fractures during the six-year post-intervention period was estimated to 14 (0.44 per 1,000 person-years). The Markov model estimated a difference in societal costs between an individual that experiences a first year hip fracture and an individual that avoids a first year hip fracture ranging from 280,000 to 550,000 SEK, and between 1.1 and 3.2 QALYs (quality-adjusted life-years, discounted 3%), for males and females aged 65–79 years and 80+ years. The cost-effectiveness analysis resulted in zero net costs and a gain of 35 QALYs, and the do-nothing alternative was thus dominated.

**Conclusion:**

The community-based elderly safety promotion program aiming at preventing accidental falls seems as cost-effective as osteoporosis pharmaceuticals.

## Background

Elderly injuries, in particular hip fractures, constitute a considerable public health problem, particularly in countries with an aging population. The risk for a woman aged 50 years to suffer from a hip fracture during her remaining lifetime is reported to exceed 15% in ten countries (of 22 investigated). The lifetime risks among men are lower, but exceed 5% in eleven countries [1]. The high incidence involves high costs, amounting to approximately 0.6% of the total health care costs in eight countries investigated during the 1990s [2]. However, the incidence in Sweden is among the highest in the world, with a remaining risk of nearly 30% for a 50-year old women and 13% for a man [1], with costs amounting to 2–3% of the total health care costs [2,3].

The issue has thus raised health policy interest in Sweden, but mostly focussing on one of the causal factors behind elderly injuries; osteoporosis (frail bones due to low bone mass). A large number of osteoporosis pharmaceuticals, ranging from food supplements (calcium and vitamin D) to specialist clinician-administrated injections, have been introduced on the Swedish pharmaceutical market and the prescription rates are high. Several pharmaceuticals have also been deemed cost-effective by the Swedish Pharmaceutical Benefits Board (LFN), and are consequently subsidized by the drug benefit system.

Somewhat simplifying the aetiology, elderly injuries are, however, caused by two necessary and concurrent factors: osteoporosis and falls [4,5]. The accidental falls, and the possibilities of preventing these and similar accidents, have received considerably less attention in Swedish health policy, and elsewhere [6]. There are, however, examples of successful fall prevention programs, from Sweden and other countries [7-9], although doubted recently [10], and there are also reports of cost-effective programs, both internationally [11,12] and from Sweden [13].

The aim of this study is thus to investigate whether a non-pharmaceutical community-based safety promotion program, which targets accidental falls, could be considered cost-effective.

## Methods

### Design

The study is performed on an implemented program, and based on data from an effect evaluation with a quasi-experimental time series analysis with several control areas. The long-term effects of the program are estimated in a health economic model, a so-called Markov model. The economic evaluation is a cost-utility analysis, i.e. it uses the health effects QALYs (quality-adjusted life-years), with a societal perspective. The analysis is performed in accordance with recommendations from the Swedish Pharmaceutical Benefit Board on economic evaluations [14].

All costs are expressed in SEK (Swedish krona) during the year 2004 (1 Euro = 9.13 SEK), converted by the consumer price index. All costs and health effects are discounted by 3% annually. The comparison alternative is the do-nothing alternative. The study was approved by the Ethics committee at Karolinska Institute North at Karolinska Hospital, 02-379.

### The program

The community-based elderly safety promotion program, Safe Seniors in Sundbyberg, was implemented during five years, 1995–1999, in a community in the Stockholm metropolitan area, Sweden. The municipality had a population of around 5,500 aged 65+ (65 years and older) in 1995, 18% of the total population, of which around 1,400 were aged 80+ years. The first hip fracture incidence during the years 1990–1995 was 12.5 per 1,000 person-years for females and 7.8 for males.

The project organization was based on principles for community organization and intersectoral collaboration. The organization included a full-time project coordinator, a steering group, containing executives from the regional health care management and the municipal elderly care organization, as well as a reference group, in which local representatives from public organizations, business companies and several voluntary organizations participated. The program was initiated by the regional health care administration that funded the project with 2.5 million SEK over five years.

The program combined structural changes in the environment with individually based measures for the elderly, using both safety promotion and injury prevention methods. Some activities were initiated for the elderly, such as lecture series on measures to increase safety (14 groups with more than 600 participants including participants from three immigrant groups), balance exercises in group (around 100 participants in collaboration with physiotherapists from the local health care and the municipal seniors' accommodations), qigong and other suitable physical activities (more than 200 participants every term, as well as free qigong in public parks during summer), and an annual outdoor fair (200–300 attendants every year, in collaboration with a large number of local organizations). Other activities focussed on environmental safety and included home visits (nurses and physiotherapists with a check-list on injury hazards issued recommendations on suitable devices), safety rounds in neighbourhoods (six rounds annually that documented injury hazards, in most cases attended to by the municipality), new routines in housing reconstructions (a formed Housing group with representatives from the municipality, the largest housing company, and the tenants' voluntary organization inspected buildings six times annually and recommended new building norms) as well as monitoring of occurred falls in seniors accommodation [15].

### The program costs

The program costs seek to include all changes in resource consumption incurred by the program, including inputs from collaborating organizations and the target group. The resource use was collected prospectively, during the intervention period, by document analyses, self-reports by key persons in collaborating organizations, and in cooperation with the project leader. As some program costs could not be estimated, e.g. safety measures taken after home visits, and other costs are possibly underestimated, in particular time consumed by the target group and some collaborating organizations, a sensitivity analysis is performed with 25% increased program costs.

Standards are used to quantify some of the resource consumption, such as 3 hours for each meeting. The running costs, i.e. consumption of telephone, office supplies, etc, are based on the hours of project work, with an assumed value of 20% of the wage cost. Some standard valuations are also used, such as 200 SEK for a meeting room. Wage costs for the project leader are taken from the project accounts. Wage costs for personnel employed by collaborators are estimated by occupation in seven different categories, including payroll taxes of 40%. The wage costs per hour vary between 310 SEK (for politicians, executives and GPs) to 70 SEK (office assistants), with the majority of the wage costs being around 150 SEK per hour. The time costs for unpaid voluntary workers as well as the time used by the target group are valued at the frequently used Swedish valuation of leisure time of 35% of average wages [16], 35 SEK per hour.

### The effect evaluation

An effect evaluation was performed to determine the number of hip fractures avoided because of the intervention, reported in detail elsewhere [17]. The design was a quasi-experimental time-series analysis with six control areas. Four control areas was chosen based on a cluster analysis on factors reported relevant for hip fracture incidence, supplemented with two larger areas. The time series analysis employed data from the years 1990 to 2001, where the years 1990–1995 were regarded as the pre-intervention period, and the years 1996–2001 were deemed the post-intervention period. The effects were expected to accumulate during this last six-year period.

The hip fracture rates, defined as ICD-diagnoses 820–820.9 (ICD-9) and S720–S722 (ICD-10), were obtained from the national Swedish Hospital Discharge Register. The study group was divided into females and males, and into two age groups, 65–79 and 80+ years. In the analysis, there were thus 28 different groups (panels); 1 intervention area+6 control areas*2 genders*2 age groups.

The panels enabled a longitudinal statistical analysis, which considered the in-group and the between-group variations during the time period investigated. The analysis gave predicted rates in the post-intervention period for the differing panels, i.e. control area-, age group- and gender-specific predicted rates. These were then applied to the Sundbyberg population, to arrive at the predicted numbers of hip fractures in Sundbyberg had the situation been the same as in the control areas. These predicted numbers were then compared with the observed numbers in Sundbyberg, resulting in an accumulated difference during the six-year post-intervention period. As the predicted numbers for Sundbyberg differ according to control area, the median difference between predicted and observed numbers for each age and gender group was considered as the outcome of the program.

### Markov model structure

The outcome of the intervention is the number of avoided first hip fractures in the intervention area after the intervention started in 1995/96 and in the following six years. Several interpretations of that outcome are possible, but we employ the conservative assumption that the avoided hip fractures are only avoided for one year, after which the individuals run the risk of contracting a hip fracture during the following years. An optimistic interpretation would be that the fractures are avoided altogether, while a pessimistic interpretation would be that all individuals contract a hip fracture in the following year.

To estimate the longer-term effects of the one-year avoided hip fractures in terms of future hip fractures and mortality as well as societal costs and health effects, a simulation model is constructed. The model data and assumptions are detailed in a technical report [18].

**Figure 1:**
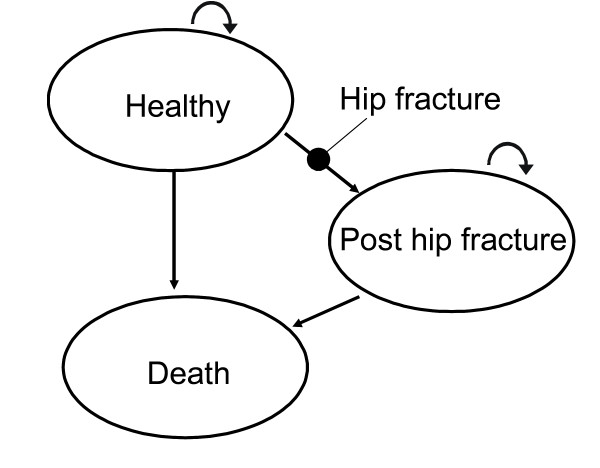
Overview of Markov model.

The model, a Markov model [19] for Monte Carlo simulation constructed in Treeage Pro (Treeage Inc.), contains three health states; Healthy, Death and Post hip fracture. The event hip fracture is modelled as a transition between the health states Healthy and Post hip fracture, with the event-specific costs included as transitional costs. Death can occur in the health states Healthy and Post hip fracture, see Figure 1. As the death risks and the cost data did not distinguish between first and subsequent hip fractures, only the first hip fracture is modelled, and the health state Post hip fracture thus contains all health and cost consequences following a first hip fracture, apart from the medical care costs incurred the first months after a hip fracture (that were regarded as hip fracture event-specific, see below).

The two age groups are reflected by different starting ages, the class middle 72 years for the age group 65–79 years and 85 years for the age group 80+ years. The simulations continue until the age of 100 years, after which no further health and cost consequences are included. The model results are obtained by comparing two rounds of simulations, where the hip fracture risk during the first year of simulation is set at 1 and 0, respectively. The individuals who avoided a first year hip fracture run the risk of contracting a hip fracture during the remaining years. The result obtained is the mean difference in costs and health consequences for individuals in the respective age and gender group with a first year hip fracture, in comparison with those that avoid a first year hip fracture.

**Figure 2:**
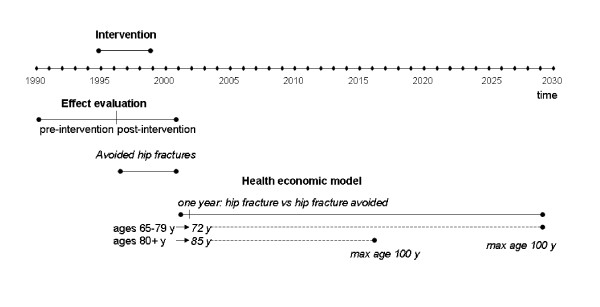
Evaluation design.

An overview of the evaluation design is found in Figure 2. The intervention was implemented during 1995–1999. The effect evaluation use data from 1990 to 2001, divided into pre-intervention period 1990–1995 and post-intervention period 1996–2001. The avoided hip fractures are assumed to accumulate during the post-intervention period. For the health economic model, the avoided hip fractures are assumed to be avoided for one year. The model estimates the differences in costs and health effects for individuals that contract a first hip fracture during the first year, with individuals that avoid a first hip fracture during the first year. The individuals aged 65–79 years are assumed aged 72 years in the model, and individuals aged 80+ are assumed aged 85 years. The cost and health differences accumulate until the individuals reach the age 100 years, or die.

### Model data

The model only contains Swedish data, see Table 1. The risk of hip fracture is the age group- and gender-specific average annual risk in the post-intervention period, which assumes that the decreased risks from the intervention are maintained in the future. The age- and gender-specific average annual mortality risks are taken from the national death register. The annual mortality risks after a hip fracture are age- and gender-specific, and divided into risks during the first year and the second and following years after a hip fracture (pers.com. F Borgström, Stockholm Health Economics, 2006-11-17). The death risks for men older than 90 years are assumed the same as for those aged 90. Contrary to other models [e.g. [20]], the excess mortality after hip fractures is not adjusted to separate the deaths directly attributed to hip fractures. The reason is that the current model does not distinguish between hip fracture-related and non-hip fracture-related deaths in terms of costs, life-years or quality-of-life. All hip fracture-related costs (except the medical treatment costs, see below) and the decrease in quality-of-life (QoL) following a hip fracture are taken from the same study [21], while the average age group- and gender-specific QoL are taken from another study [22]. For individuals experiencing a hip fracture, the average decrease in QoL is deducted from the average age group- and gender-specific QoL, which results in age group- and gender-specific QoL after a hip fracture. The 95% confidence intervals of costs are used within the model, modelled as a uniform distribution.

**Table 1:** Model data. Costs in SEK 2004 (95% confidence intervals in parenthesis).

**Type of data**	**Group**	**Estimate**	**Source**
Annual risk of hip fracture	men 65–79 y	0.0049	Effect evaluation
	men 80+ y	0.0175	
	women 65–79 y	0.0053	
	women 80+ y	0.0261	
Death risks after hip fracture	1^st ^year after hip fracture: 2^nd ^and following years after fracture: age- and gender-specific		pers.com. F. Borgström, Stockholm Health Economics, 2006-11-17
Death risks	age- and gender-specific		National Death Register
Health care costs	initial cost	110,364 (105,123 – 115,221)	Stockholm County Council healthcare data base
	annual cost	12,590 (11,141 – 14,243)	
Community care	annual cost	38,200 (26,304 – 55,711)	[21]
Pharmaceuticals	annual cost	1,552 (1,360 – 1,753)	[21]
Informal care	annual cost	3,223 (1,954 – 5,524)	[21]
QoL reduction from hip fracture	annual	0.17	[21]
Average QoL	men 65–69 y	0.78	[22]
	men 70–79 y	0.76	
	men 80+ y	0.68	
	women 65–69 y	0.75	
	women 70–79 y	0.66	
	women 80+ y	0.57	

The hip fracture-related medical care costs are taken from a set of databases that record all medical care provided to the population of Stockholm County (inhabitants in 2004 1.9 million), (see [18] for details). The databases include in-patient, out-patient and primary care episodes, as well as a population register. The in-patient and out-patient health care costs are based on the County s DRG (Diagnosis Related Groups) price system. Primary care is not included in the DRG system, which is why a standard cost [23] is used for care episodes registered with GPs, nurses or physiotherapists.

The medical care costs used are based on all patients aged 65+ years with a hip fracture operation (code NFJ00 – NFJ99) during the year 2002. The initial costs are average costs per individual accumulated during 6 months after the first hip fracture operation in 2002, for individuals that had no hip fracture operation in 2001 and survived these first 6 months. All medical care costs during the time period are included, thus assuming that they are related to the hip fracture. The annual costs are the annual average costs for the patients during the years 2003–2005, i.e. three years after the first hip fracture. In-patient costs are only included if hip fracture is a recorded diagnosis, all but obviously hip fracture unrelated out-patient costs (such as dialysis and cancer treatments) are included, and all primary care episodes are included. The medical care costs during the year of death could have been included, as transitional costs in the model, but they are excluded as they are probably similar to the costs for individuals in the same ages without hip fractures. The medical care costs are mean costs over the gender and age groups, adjusted to the 2004 price level by the consumer price index. The 95% confidence interval (CI) is obtained with a bootstrap simulation, performed in the SAS statistical program (SAS Institute, 2007), and using the percentile method [24].

### Sensitivity analyses

To investigate the uncertainty in the cost-effectiveness ratio, a number of sensitivity analyses are performed. All model parameters are altered in univariate and multivariate analyses based on alternative data sources (details are found in [18]). The overall model uncertainty is investigated in a bootstrap analysis based on the model simulation results, with a 95% confidence interval calculated by the percentile method [24]. Program-specific analyses include alternative assumptions on the costs and the effectiveness of the program. The sensitivity of the cost-effectiveness ratio to the program effectiveness is based on alternative estimates from the effect evaluation on the numbers of first hip fractures avoided in the intervention area. The lowest effectiveness is based on the result from the control area that gives the lowest number of avoided hip fractures in the intervention area (0 for men in both age groups, -14 for women aged 65–79, and +15 for women aged 80+ years). This is actually an increase in the number of hip fractures because of the intervention, and thus a very conservative assumption. The highest effectiveness is based on data taken from the control area that results in the highest number of avoided hip fractures in the intervention area (-1 for men aged 65–79, -26 for women aged 65–79, -3 for men aged 80+, +9 for women aged 80+ years). The effect on the cost-effectiveness ratio from program costs is investigated by increasing them by 25%. Finally, a break-even analysis investigates the required number of avoided hip fractures needed to consider the intervention to be very cost-effective in Sweden, i.e. with a cost per QALY below 100,000 SEK [25].

## Results

### Effect evaluation

The effect evaluation resulted in decreased numbers of hip fractures among women and men aged 65–79 years and among men aged 80+ years in Sundbyberg as compared to the control areas. The median number of avoided hip fractures among the control areas was 8 for women 65–79 years old and 3 each for the men in the two age groups. For women older than 80 years, however, the estimates indicated an increase in Sundbyberg as compared to the control areas. The sole reason was the very high number of hip fractures in one year (41 in year 2000), in comparison with previous and later years (between 26 and 29 during the rest of the post-intervention period) [17]. It is unlikely that the high number during one year is due to the intervention, but instead of adjusting the figure, e.g. by replacing it with the average number during the period, we conservatively concluded that the intervention had not had any effect among the elderly women. The result of the effect evaluation was thus interpreted as a total of 14 avoided hip fractures, with 8 among women aged 65–79 years and 3 each for the men, and no effects among women aged 80+. In the sensitivity analyses on program effectiveness the increase among women aged 80+ is included.

### Model estimates

The model estimates show a difference in societal cost between an individual with a first year hip fracture vs. an individual that avoids a first year hip fracture ranging from 280,000 to 550,000 SEK in the age and gender groups, with the largest differences in community care costs, see Table 2. The differences in health per individual are estimated to between 1.1 and 3.2 QALYs (discounted 3%), and 0.9 to 4.2 life-years (YLS, undiscounted). The differences are larger for the younger age groups, with the largest differences in costs for women and in health effects for men.

**Table 2:** Model estimates. Costs in SEK 2004.

		**Total cost of which:**				Health effects*		
		**Medical care**	**Pharmaceuticals**	**Community care**	**Informal care**	**QALYs^§^**	**YLS**	**YLS^§^**

Men, aged 65–79 y								

1st year	466,630	177,121	9,730	256,387	23,392	3.54	20.48	12.51
avoided 1st year	30,160	13,256	568	14,974	1,363	6.74	16.31	9.42
*difference*	*436,469*	*163,865*	9,162	*241,414*	*22,028*	*3.20*	*4.17*	*3.09*

Men, aged 80+ y								

1st year	296,974	140,625	5,242	138,488	12,620	1.72	11.21	8.55
avoided 1st year	19,918	10,118	330	8,684	787	3.03	9.89	7.41
*difference*	*277,056*	*130,507*	4,911	*129,804*	*11,833*	*1.32*	*1.31*	*1.14*

Women, aged 65–79 y								

1st year	608,211	207,675	13,474	354,703	32,358	3.93	17.11	10.10
avoided 1st year	60,516	24,246	1,220	32,121	2,929	6.63	13.89	7.81
*difference*	*547,695*	*183,429*	12,255	*322,582*	*29,429*	*2.70*	*3.22*	*2.29*

Women, aged 80+ y								

1st year	370,170	156,440	7,181	189,304	17,245	1.85	9.71	7.30
avoided 1st year	39,846	18,491	720	18,901	1,734	2.97	8.85	6.57
*difference*	*330,324*	*137,948*	6,462	*170,403*	*15,511*	*1.12*	*0.86*	*0.73*

*survivors at the age of 100 years are assumed to be dead at the age of 100.

§ discounted 3%.

### Cost-effectiveness analysis

The cost-effectiveness analysis is summarized in Table 3. Total societal program costs amount to 6.45 million SEK, of which 4.4 million SEK consist of time costs; for the wages for employed by the project (11,200 hours), for those employed by other organizations (11,100 hours), for volunteers (2,200 hours) and for participants (18,300 hours). The remainder consists of running costs, other costs (such as reconstruction costs, devices, meeting rooms, etc) and some participants' outlays.

**Table 3:** Summary of the cost-effectiveness analysis. Costs in SEK 2004.

	**Cost item**	**Total**
Program costs		

Project wage costs	1,783,889	
Other wage costs	1,920,119	
Volunteers	76,025	
Running costs	308,959	
Other costs	1,527,409	
Participants' costs	834,748	
*Total Program costs*		*6,451,149*

Costs avoided		

Medical care	2,350,551	
Pharmaceuticals	140,257	
Community care	3,694,309	
Informal care	337,017	
*Total Costs avoided*		*6,522,134*

*Net costs*		*-70,985*

Health effects		

Life-years saved (YLS)*		42.23

QALYs		35.16

*undiscounted

When the model estimates are applied to the number of individuals that the effect evaluation indicated had avoided a hip fracture for one year, i.e. 8 women aged 65–79 years and 3 men in each of the age groups, the total costs avoided amount to 6.52 million SEK. As the intervention costs amounted to 6.45 million SEK, the net costs thus become a saving of 71,000 SEK, i.e. close to 0. The total health effects in QALYs amount to 35, and to around 42 YLS (life-years, undiscounted). As the net costs are negative, the comparison treatment, the do-nothing alternative, is dominated with higher costs and lower QALYs.

### Sensitivity analyses

The cost-effectiveness ratio is most sensitive to the assumption on no costs and health effects after the first year following a hip fracture, as the two multivariate analyses using the assumption (I and J) result in costs above 200,000 SEK per QALY, see Table 4. Analysis J actually describes another condition (i.e. one that is rather common but not very serious), which is considered to be the most conservative assumption possible. Among the univariate analyses, only the inclusion of costs during added life-years (analysis E) results in costs per QALY above 100,000 SEK. Three more analyses indicate very low positive costs, while the remainder results in negative net costs. Among the program-specific sensitivity analyses, only the lowest program effectiveness, which implied a net increase of 1 fracture, leads to considerable changes, i.e. to a cost per QALY of around 180,000 SEK. The break-even analysis shows that the program needs to avoid only 4 and 5 hip fractures among males and females aged 65–79 years, respectively, to obtain a cost per QALY below 100,000 SEK.

**Table 4:** Sensitivity analysis results. Costs in SEK 2004.

	**Costs avoided**	**Net costs**	**QALYs**	**Cost per QALY**
Base case	6,522 134	-70,985	35	<0

Model parameters				

A. Fracture risk [39]	6,479,356	-28,207	35	<0
A. Fracture risk doubled	5,978,641	472,508	33	14,327
B. Mortality risk [40]	7,401,024	-949,875	29	<0
C. Medical treatment costs [21]	11,793,758	-5,342,609	35	<0
C. Medical treatment costs, average difference	6,420,258	30,891	35	879
1 year before and 2 years after hip fracture				
D. 2^nd ^and following year costs [21]	6,709,090	-257,941	35	<0
E. Non-market productivity incl. [41]	6,789,172	-338,023	35	<0
E. Costs in added life-years [14]	447,200	6,003,949	35	170,907
F. Alternative QoL weights [42]	6,522,134	-70,989	48	<0
G. Discount rate 0%	7,535,125	-1,083,976	45	<0
G. Discount rate 5%	5,986,099	465,050	30	15,313
H. Mortality and hip fracture risks [39,40]	7,385,325	-934,176	29	<0
I. No costs or QoL effects after 1st year	1,935,529	4,515,620	22	202,041
J. Another disease; fracture risks doubled, no costs or QoL effects after 1st year	1,730,899	4,720,250	21	228,805

Program specific				

Program costs +25%	6,522,134	1,541,798	35	43,888
Program effectiveness: lowest	2,712,870	3,738,279	21	178,013
Program effectiveness: highest	12,534,792	-6,083,643	67	<0

**Figure 3:**
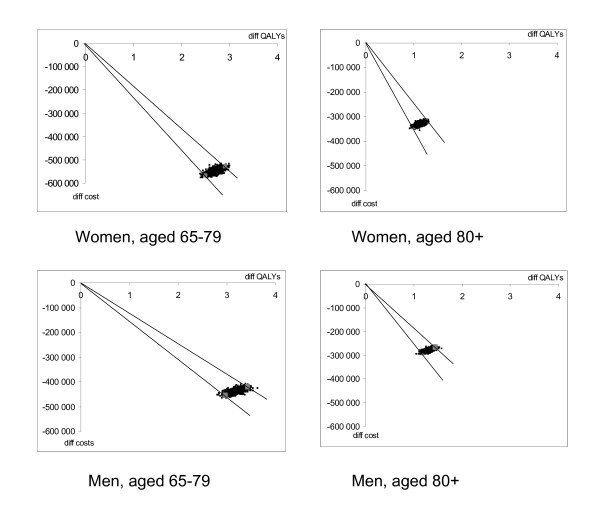
Bootstrap simulation of model estimated differences in cost and QALYs. Costs in SEK 2004.

The bootstrap estimates, performed on the model simulation results, are very condensed, see Figure 3, with narrow 95% confidence intervals and replicates spread around the base case estimates on differences in costs and QALYs between the hip fracture groups. The uncertainty is more pronounced in the differences in QALYs, but for no group is the confidence interval wider than 1 QALY.

## Discussion

The elderly safety promotion program was estimated to result in 14 avoided hip fractures during six years after the initiation of the program. These 14 avoided hip fractures, assuming they were avoided for one year and the individuals run the risk of contracting a hip fracture during subsequent years, result in zero net costs and an increase in health of 35 QALYs, in comparison with a do-nothing alternative. All sensitivity analyses, including some based on very conservative assumptions, give costs per QALY below 250,000 SEK, which is considered moderately cost-effective in Sweden [25]. Furthermore, a break-even analysis shows that 9 hip fractures avoided (4 among men and 5 among women aged 65–79 years) are sufficient to obtain a cost per QALY below 100,000 SEK, which is considered very cost-effective in Sweden. The program is very likely to be cost-effective, and should thus be implemented on a large scale.

The result of the cost-effectiveness analysis hinges on a number of evaluation design choices, which in turn leads to choices of data sources, methodologies and assumptions. The effectiveness of the intervention was measured as number of first hip fractures among inhabitants in different geographical areas, which might underestimate the effects of the intervention. The design of the effect evaluation is deemed appropriate for community-based programs, i.e. a quasi-experimental design with several control areas [26], combined with an elaborate time-trend analysis [27]. The accuracy of the effect evaluation is difficult to ascertain, but the result might be compared with previous studies to judge plausibility. To be able to estimate the long-term effects on societal costs and health consequences due to the avoided hip fractures, a health economic model was used. The model and the results might be compared with similar models, to judge the accuracy of the estimates. Modelling requires an assumption on when the avoided hip fractures should have had occurred. The choice made was that all hip fractures are avoided during the same year, and only avoided for one year, after which the individuals run the risk of contracting a hip fracture during coming years. Alternative assumptions are more optimistic or pessimistic, but as the cross-sectional data does not allow for individual-level follow-up, the true relationship cannot be established. The risk of hip fractures during remaining years was however taken from the average risk in the intervention area during the post-intervention period, which might overestimate the cost-effectiveness somewhat, as the lower hip fracture rates after the program are assumed maintained in the future.

The effect evaluation data is taken from a register on hospital patients according to residence and not location of injury, which might underestimate the true effects of the program. The program measures that aimed at structural changes in the environment might have prevented hip fractures also among visiting non-residents of Sundbyberg. The individually based measures would have prevented injuries also when the persons are outside Sundbyberg. However, if residents that have taken part in the intervention move out, and new residents that have not taken part move in, the program effects would be diluted. The migration aspect calls for a rather short follow-up period, like the present study's, even though some program measures will probably affect the hip fracture incidence during several years after the end of the follow-up period.

Community-based interventions require more elaborate evaluations and statistical methods than controlled trials measuring individual-level outcomes [28-30]. However, the intervention area and several of the control areas are small geographical areas, which increase the risk that mere chance affects the estimates. The effect evaluation data was however analysed in a longitudinal analysis, that seeks to take account of both within-area, between-area and population-group variation. The accuracy of the effect evaluation is nevertheless difficult to ascertain, in particular as one extreme value managed to alter the estimates for one panel altogether (women aged 80+).

The effect evaluation result of an accumulated decrease of 14 hip fractures during the six-year post-intervention period is equivalent to one quarter of the 60 hip fractures that occurred in the intervention area the year before the program. This seems to be in line with the results from a review on population-based interventions for the prevention of fall-related injuries in older people [8], which stated that all five included studies reported decreases of fall-related injuries, of the magnitude 6 to 33%. Furthermore, a break-even analysis is included among the sensitivity analyses, to enable judgement of the plausibility that the intervention is cost-effective, showing that such a small number as 9 avoided hip fractures is sufficient to reach a cost-effective result.

A health economic simulation model is necessary to obtain results that would be considered cost-effective, as only the shorter term effects, i.e. during one year, would have resulted in costs per QALY of about 2.3 million SEK (data not shown), which is not considered cost-effective in Sweden. However, the health economic modelling of effects after follow-up is common and indeed recommended [14,19] to obtain a full measurement of the effects of medical interventions.

The Markov model is fairly simple, including few data items and only hip fracture-related risks and costs, taken from a small number of previous studies, which increase the internal validity. The external validity is enhanced by the fact that only Swedish data is used. The model data is mainly taken from secondary data sources, while the program data is taken from an implemented program, and is thus primary data. The cost data employed in the model might be underestimated, due to difficulties to collect and value all hip-fracture related costs. The medical treatment costs, for example, do not include costs for home visits made by medical personnel, very frequent for the patient group, due to lack of reliable cost estimates of the type of care.

Comparison with previously published Swedish osteoporosis models is hampered by the differing objectives of the models; this model estimates the differing costs and health effects for individuals with and without a hip fracture during the first year, while other models are based on clinical trials, where the effects of changing fracture risks are estimated. Costs during the 2^nd ^and following years after hip fracture are however handled differently in the present and previous studies [e.g. [20,31-34]]. The present study only models first hip fractures which is why the costs for subsequent hip fractures are assumed to be included in the Post hip fracture state. The study population from which the costs and health effects are taken includes both individuals with a first hip fracture and individuals with previous hip fractures [20], which is the reason why that use of the data seems appropriate. However, this implies that the reported costs are also used for the 2^nd ^and following years, where most, but not all [34], previous studies have instead assumed that the long-term annual costs only consist of nursing homes stays for a proportion of the patient group. In one sensitivity analysis (analysis D), these alternative costs in the 2^nd ^and following years give somewhat smaller cost differences for the younger age group, but actually double the cost differences for those aged 80+ years.

For comparison purposes, there is a reference model available (at the International Osteoporosis Foundation, IOF, homepage) where relevant parameters can be entered to obtain results in terms of cost and QALY differences between treated and untreated patients [35,36]. Even though it is not possible to adjust the parameters to fully resemble the model of this study, a 100% risk reduction in hip fractures during 1 year for women aged 72 years gives costs of around 112,000 SEK until the age of 87 years, to be compared with the present model's estimate of 60,000 SEK until the age of 100 years. The difference in costs between first year hip fracture and avoided first year hip fracture for women aged 72 years would have been around 700,000 SEK in the reference model, as compared to 550,000 SEK in the present model. For women aged 85 years, the reference model also estimates higher costs. The estimated QALY differences for both age groups are also higher in the reference model. Compared to the IOF reference model, the present model thus estimates lower costs but also somewhat lower QALYs. There is no IOF reference model for men.

The societal perspective is the only appropriate perspective for an analysis of a community-based program, as the pronounced goal of the program is to mobilise whole communities, through collaborating organizations and changes in life style in the target group. The difficulties in collecting the true costs, given self-reports of a large number of collaborators, might however lead to underestimates. However, the costs were collected prospectively, and reported to and discussed with the collaborators annually. The largest underestimates might therefore be found for the participant's time consumed in safety promotion measures, as only time spent in measures taken within the program could be included. The cost-effectiveness analysis is not very sensitive to the program costs, however; the analysis that increased these costs by 25% resulted in costs per QALY below 100,000 SEK.

The cost-effectiveness might, on the other hand, be underestimated, due to positive externalities from the program. One such externality stems from the changes in the physical environment, such as building norms and swift removal of street hazards, which potentially also affect injuries among other population groups. Another externality is the probable health-enhancing effects of some of the individually based measures; an increased social network as well as increased physical activity and improved dietary habits. Finally, the analysis only includes prevented hip fractures, which is an underestimate given that a reduced incidence of hip fractures probably implies reductions also in other fractures and injuries [37].

The overall result of the program might appear modest; 14 avoided first hip fractures that lead to 35 QALYs. As the estimated costs avoided balanced the intervention costs, these QALYs were gained at no cost. It is thus a waste of societal resources not to implement similar programs. This contradicts the fear that high intensity interventions might be too costly to be considered cost-effective [10]. However, the avoided hip fractures only constitute a decrease of 0.44 per 1,000 person-years, to be compared with the average hip fracture incidence in Sweden of around 11 per 1,000 in the ages 65+ years. That means that around 1 of 25 hip fractures potentially could be avoided by the program, if implemented on a national level. This is no dramatic decrease, but translates into 700 individuals in Sweden that could avoid a hip fracture each year. This is of course due to the high Swedish hip fracture incidence, which also increases the cost-effectiveness of the intervention together with the considerable societal costs for each hip fracture. The generalizability of the cost-effectiveness analysis might therefore be restricted to countries with similar characteristics.

Finally, the program described here requires commitment from a large number of collaborators in the local community, which might be difficult to achieve and sustain for an extended time period. The incentives for the collaborating organizations to mobilise their resources for such programs might be few [38], which might render the implementation of similar programs difficult.

## Conclusion

This cost-effectiveness analysis shows that the community-based elderly safety promotion program is very likely to be cost-effective, and similar programs should thus be encouraged. As the analysis conforms to the recommendations issued by the Swedish Pharmaceutical Benefits Board and uses similar methods and data sources as analyses of osteoporosis pharmaceuticals submitted to and deemed cost-effective by the Board, the community-based elderly safety promotion program is as cost-effective as osteoporosis pharmaceuticals.

## Competing interests

No author has any financial or non-financial competing interests in relation to the study reported.

## Authors' contributions

PJ initiated and designed the study, acquired, analysed and interpreted the data, and drafted the manuscript. PT contributed to the conception and design of the study, and revised the manuscript. SS contributed to the design and data acquisition of the study, and revised the manuscript. CR contributed to the design of the study and interpretation of data, and revised the manuscript. All authors read and approved the final manuscript.

## References

[B1] Kanis JA, Johnell O, de Laet C, Jönsson B, Odén A, Ogelsby AK (2002). International variations in hip fracture probabilities: Implications for risk assessment. J Bone Miner Res.

[B2] SBU (The Swedish Council on Technology Assessment in Health Care) (2003). Osteoporos – prevention, diagnostik och behandling [Osteoporosis – prevention, diagnostics and treatment] Stockholm.

[B3] Borgström F, Sobocki P, Ström O, Jönsson B (2007). The societal burden of osteoporosis in Sweden. Bone.

[B4] Svanström L (1999). Ageing and safety promotion – what do we know and where are we going?. Promot Educ.

[B5] Vestergaard P, Rejnmark L, Mosekilde L (2001). Hip fracture prevention. Cost-effective strategies. Pharmacoeconomics.

[B6] Järvinen TL, Sievänen H, Khan KM, Heinonen A, Kannus P (2008). Shifting the focus in fracture prevention from osteoporosis and falls. BMJ.

[B7] Gillespie LD, Gillespie WJ, Robertson MC, Lamb SE, Cumming RG, Rowe BH (2003). Interventions for preventing falls in elderly people. Cochrane Database of Systematic Reviews.

[B8] McClure R, Turner C, Peel N, Spinks A, Eakin E, Hughes K (2005). Population-based interventions for the prevention of fall-related injuries in older people. Cochrane Database of Systematic Reviews.

[B9] Lindqvist K, Timpka T, Schelp L (2001). Evaluation of an inter-organizational prevention program against injuries among the elderly in a WHO Safe Community. Public Health.

[B10] Gates S, Fisher JD, Cooke MW, Carter YH, Lamb SE (2008). Multifactorial assessment and targeted intervention for preventing falls and injuries among older people in community and emergency care settings: systematic review and meta-analysis. BMJ.

[B11] Salkeld G, Cumming RG, O'Neill E, Thomas M, Szonyi G, Westbury C (2000). The cost effectiveness of a home hazard reduction program to reduce falls among older persons. Aust N Z J Public Health.

[B12] Campbell AJ, Robertson MC, La Grow SJ, Kerse NM, Sanderson GF, Jacobs RJ, Sharp DM, Hale LA (2005). Randomised controlled trial of prevention of falls in people aged ≥75 with severe visual impairment: the VIP trial. BMJ.

[B13] Ferraz Nunes J, Ader M (2005). Förebyggande av höftfrakturer i Skaraborg [Prevention of hip fractures in Skaraborg].

[B14] LFN (Swedish Pharmaceutical Benefits Board) General guidelines for economic evaluations from the Pharmaceutical Benefits Board (LFNAR 2003:2).

[B15] Sadigh Andersson S, Hökby A (2000). Säkra Seniorer i Sundbyberg – Slutrapport med utvärdering [Safe Seniors in Sundbyberg – final report with evaluation].

[B16] Claesson L, Gosman-Hedström G, Johannesson M, Fagerberg B, Blomstrand C (2000). Resource utilization and costs of stroke unit care integrated in a care continuum: A 1-year controlled, prospective, randomized study in elderly patients. Stroke.

[B17] Johansson PM, Ponce de Leon A, Sadigh S, Tillgren PE, Rehnberg C (2008). Statistical modelling needed to find the effects from a community-based elderly safety promotion program.

[B18] Johansson P (2008). A cost-effectiveness model on avoided hip fractures Technical report Stockholm.

[B19] Kuntz KM, Weinstein MC, Drummond M, McGuire A (2001). Modelling in economic evaluation. Economic evaluation in health care – Merging theory with practice.

[B20] Borgström F, Jönsson B, Ström O, Kanis JA (2006). An economic evaluation of strontium ranelate in the treatment of osteoporosis in a Swedish setting. Osteoporos Int.

[B21] Borgström F, Zethraeus N, Johnell O, Lidgren L, Ponzer S, Svensson O, Abdon P, Ornstein E, Lunsjö K, Thorngren KG, Sernbo I, Rehnberg C, Jönsson B (2006). Costs and quality of life associated with osteoporosis-related fractures in Sweden. Osteoporos Int.

[B22] Burström K, Johannesson M, Diderichsen F (2001). Health-related quality of life by disease and socio-economic group in the general population in Sweden. Health Policy.

[B23] Swedish Association of Local Authorities and Regions (Sveriges Kommuner och Landsting) Verksamhet och ekonomi i landsting och regioner [Activities and economy in county councils and regions] Stockholm.

[B24] Briggs AH, Drummond M, McGuire A (2001). Handling uncertainty in economic evaluation and presenting the results. Economic evaluation in health care – Merging theory with practice.

[B25] Swedish National Board of Health and Welfare (Socialstyrelsen) (2004). Guidelines for cardiac care, 2004 Stockholm.

[B26] Cummings P, Koepsell TD (2002). Statistical and design issues in studies of groups. Inj Prev.

[B27] Cook TD, Campbell DT (1979). Quasi-experimentation – Design & analysis issues for field settings.

[B28] Nutbeam D (1998). Evaluating health promotion – progress, problems and solutions. Health Promot Int.

[B29] Bauman A, Koepsell TD, Brownson RC, Petitti DB (2006). Epidemiologic issues in community intervention. Applied epidemiology.

[B30] de Leon AP, Svanström L, Welander G, Schelp L, Santesson P, Ekman R (2007). Differences in child injury hospitalizations in Sweden: The use of time-trend analysis to compare various community injury-prevention approaches. Scand J Public Health.

[B31] Jönsson B, Hedbrant J, Juhnell O (1993). A computer simulation model to analyse the cost-effectiveness of fracture prevention of osteoporosis.

[B32] Zethraeus N, Johannesson M, Jönsson B (1998). A computer model to analyse the cost-effectiveness of hormone replacement therapy.

[B33] Willis M, Ödegaard K, Persson U, Hedbrant J, Mellström D, Hammar M (2001). A cost-effectiveness model of Tibolone as treatment for the prevention of osteoporotic fractures in postmenopausal women in Sweden. Clin Drug Invest.

[B34] Johnell O, Jönsson B, Jönsson L, Black D (2003). Cost effectiveness of alendronate (Fosamax) for the treatment of osteoporosis and prevention of fractures. Pharmacoeconomics.

[B35] Ström O, Zethraeus N, Borgström F, Johnell O, Jönsson B, Kanis J (2006). IOF cost-effectiveness reference model Background document European Health Economics.

[B36] Zethraeus N, Borgström F, Ström O, Kanis JA, Jönsson B (2007). Cost-effectiveness of the treatment and prevention of osteoporosis – a review of the literature and a reference model. Osteoporos Int.

[B37] Bergland A, Wyller TB (2004). Risk factors for serious fall related injury in elderly women living at home. Inj Prev.

[B38] Johansson PM, Eriksson L, Sadigh S, Rehnberg C, Tillgren PE (2008). Participation, resource mobilisation and financial incentives in community-based health promotion – an economic evaluation perspective.

[B39] Kanis JA, Johnell O, Odén A, Sernbo I, Redlund-Johnell I, Dawson A, de Laet C, Jönsson B (2000). Long-term risk of osteoporotic fracture in Malmö. Osteoporos Int.

[B40] Johnell O, Kanis JA, Odén A, Sernbo I, Redlund-Johnell I, Pettersson C, de Laet C, Jönsson B (2004). Mortality after osteoporotic fractures. Osteoporos Int.

[B41] Statistics Sweden (SCB) (2002). Tidsanvändnings-studien [The study on time uses].

[B42] Tidermark J, Zethraeus N, Svensson O, Törnkvist H, Ponzer S (2002). Femoral neck fractures in the elderly: Functional outcome and quality of life according to EuroQol. Qual Life Res.

